# Bevacizumab-based treatment as salvage therapy in patients with recurrent symptomatic brain metastases

**DOI:** 10.1093/noajnl/vdaa038

**Published:** 2020-03-16

**Authors:** Anna Sophie Berghoff, Michael Oliver Breckwoldt, Lars Riedemann, Kianush Karimian-Jazi, Sarah Loew, Franziska Schlieter, Julia Furtner, Marc Cinci, Michael Thomas, Moritz J Strowitzki, Frederik Marmé, Laura L Michel, Thomas Schmidt, Dirk Jäger, Martin Bendszus, Matthias Preusser, Wolfgang Wick, Frank Winkler

**Affiliations:** 1 Department of Medicine I, Medical University of Vienna, Vienna, Austria; 2 Comprehensive Cancer Center, Medical University of Vienna, Vienna, Austria; 3 Clinical Cooperation Unit Neuro-Oncology, German Cancer Consortium (DKTK), German Cancer Research Center (DKFZ), Heidelberg, Germany; 4 Department of Neuroradiology, University Hospital Heidelberg, Heidelberg, Germany; 5 Neurology Clinic and National Center for Tumor Diseases, University Hospital Heidelberg, Heidelberg, Germany; 6 Department of Biomedical Imaging and Image-guided Therapy, Medical University of Vienna, Vienna, Austria; 7 Department of Medical Oncology and Internal Medicine VI, National Center for Tumor Diseases, Heidelberg University Hospital, Heidelberg, Germany; 8 Department of Thoracic Oncology, University Hospital Heidelberg and Translational Lung Research Center Heidelberg, Heidelberg, Germany; 9 Department of General, Visceral and Transplantation Surgery, University of Heidelberg, Heidelberg, Germany; 10 National Center for Tumor Disease, Gynecologic Oncology, University Hospital Heidelberg, Heidelberg, Germany

**Keywords:** bevacizumab, brain edema, brain metastases, recurrence, steroids

## Abstract

**Background:**

Salvage treatment for recurrent brain metastases (BM) of solid cancers is challenging due to the high symptomatic burden and the limited local treatment options.

**Methods:**

Patients with recurrent BM with no option for further local therapies were retrospectively identified from BM databases. Bevacizumab-based treatment was initiated as a salvage treatment. Radiological imaging before and after bevacizumab-based treatment was reevaluated for treatment response using the Response Assessment in Neuro-Oncology (RANO) BM criteria.

**Results:**

Twenty-two patients (36.4% male) with recurrent BM from breast cancer (40.9%), colorectal cancer (31.8%), or lung cancer (27.3%) were identified. Previous BM-directed therapies were radiosurgery in 16/22 (72.7%) patients, whole-brain radiotherapy in 8/22 (36.4%), and neurosurgical resection in 11/22 (50.0%). Time since BM diagnosis to initiation of bevacizumab treatment was 16.5 months. Of 22 patients 14 (63.6%) received concurrent systemic therapies. Neurological symptom improvement could be achieved in 14/22 (63.6%) and stabilization in 6/22 (27.3%) patients, resulting in a clinical benefit in 20/22 (90.9%) patients. Steroids could be reduced or stopped in 15/22 (68.2%) patients. Rate of improvement on T1-weighted imaging was 15/19 (78.9%; median reduction: −26.0% ± 32.9) and 19/20 (95%; median reduction: −36.2% ± 22.2) on T2-weighted FLAIR imaging. According to RANO-BM best response was partial response in 7/19 (36.8%), stable disease in 9/19 (47.3%), and progressive disease in 3/19 (15.7%) patients. Median CNS-specific progression-free survival was 8 months and median overall survival after initiation of bevacizumab treatment was 17 months.

**Conclusions:**

Bevacizumab-based treatment had clinically relevant intracranial activity in the vast majority of patients suffering from recurrent, symptomatic BM. The data supports a prospective clinical trial of bevacizumab as a salvage treatment in BM.

**Key Points**

- Bevacizumab-based treatment had clinically relevant intracranial activity in the majority of patients suffering from recurrent symptomatic BM.

**Importance of the Study**

Treatment of recurrent BM with no option for further local therapies is challenging due to the high symptomatic burden and frequent steroid dependency. Here, we investigated that bevacizumab-based treatment had clinically relevant intracranial activity in the majority of patients suffering from recurrent symptomatic BM in this retrospective analysis. Given the favorable effects on neurological symptoms, the absence of unexpected side effects, the possibility to reduce steroid treatment, and the promising progression-free and overall survival, further investigation of bevacizumab as a salvage treatment in BM patients is warranted.

Brain metastases (BM) are a major burden for patients suffering from solid tumors as BM are frequently associated with relevant neurological deficits, compromised quality of life, steroid dependence, and limited life expectancy. Isolated intracranial progression is the predominant cause of death in many BM patients, ranging from 30% of patients suffering from lung cancer up to 48% of patients suffering from brain metastatic breast cancer.^1^ Local therapies including neurosurgical resection, stereotactic radiosurgery (SRS), and whole-brain radiotherapy (WBRT) are still the mainstay treatment of BM, although systemic therapies are of increasing importance especially in patients with asymptomatic BM.^2^ Upon recurrence, local treatment options are limited due to the increasing risk of radionecrosis and radiation-induced white matter leukodystrophy.^3,4^ Neurosurgical resection is a major surgical procedure and not feasible in patients with a reduced overall condition or in patients with BM in eloquent areas.^5^ Therefore, the treatment of patients with recurrent, often highly symptomatic BM disease is a major clinical challenge. Steroids are widely used to control clinical symptoms caused by perifocal edema.^2,6,7^ However, steroid treatment has quality-of-life impairing side effects, including iatrogenic Cushing syndrome, which is frequently evident already after only a few weeks of treatment.^6,7^ Steroid side effects like mood changes, metabolic derailment, sleep disorders, and myopathy add to the symptoms of advanced cancer and can further impair the quality of life. However, the steroid effect on the tumor edema is needed, as otherwise patients suffer from signs of increased cranial pressure including headache, nausea, and vomiting or focal neurological deficits. Therefore, treatment of recurrent symptomatic BM faces a dreadful vicious circle of irreplaceable steroid treatment and steroid side effects.^7^

The anti-angiogenic treatment has been widely investigated in several frequently BM causing entities such as non-small cell lung cancer, colon cancer, or breast cancer. However, none of the prospective studies could so far show a marked impact on overall survival in patients metastatic extracranial tumors without BM.^8,9^ Nevertheless, extensive neo-angiogenesis is a well-characterized hallmark of BM arguing that in the specific context of brain metastatic disease, anti-angiogenic therapy might have a particular therapeutic impact.^9–12^ Indeed, brain-specific prevention of lung cancer BM by the anti-vascular endothelial growth factor (VEGF) antibody bevacizumab was observed in preclinical models and retrospective analysis of phase III trials, further underscoring the therapeutic potential of anti-angiogenic therapies in the particular context of BM.^9,10,13^ In this study, we investigated the clinical efficacy of bevacizumab, a monoclonal antibody against VEGF, as salvage therapy in patients with symptomatic recurrent BM not eligible for further local therapies.

## Methods

### Patients

Patients with recurrent BM treated at the University of Heidelberg or the Medical University of Vienna were identified. Bevacizumab-based treatment was initiated as a salvage treatment after discussion in an interdisciplinary tumor board. Only patients with no option for local therapy, including either radiotherapy (SRS and WBRT) or neurosurgical resection, were eligible for bevacizumab-based treatment based on the previous case reports.^14,15^ Patients with clinical contraindications for bevacizumab such as prior CNS bleeding, uncontrolled hypertension, or previous bowl fistulation were not treated with bevacizumab and in consequence not included in the present analysis. For the current analysis, BM databases were used to identify the patients treated with bevacizumab for progressive BM. Bevacizumab was given either as a flat dose of 400 mg every 2 weeks, according to local standard of care for primary brain tumors (Vienna), or in a body weight-adapted dosing of 7.5–10 mg/kg body weight every 2–3 weeks (Heidelberg). Clinical data including applied treatments and survival time were retrieved by retrospective chart review.

Neurological symptoms were defined as the presence of either sign of increased intracranial pressure, neurological deficits, neuropsychological symptoms or epileptic seizures. Signs of increased cranial pressure were defined as the presence of one or more of the following: nausea, headache, or emesis. The neurological benefit was evaluated semi-quantitatively by analysis of medical records of the treating physician and defined as a reduction in neurological symptoms and the resulting improvement in the independence in the activities of daily life. Clinical benefit was defined as stabilization or improvement of neurological symptoms.

### Radiological Analysis

Magnetic resonance imaging (MRI) was performed before (baseline scan) and after the initiation of bevacizumab treatment. All MRI scans were retrieved and centrally reviewed (M.O.B. 10 years of experience, K.K.J. 5 years of experience) for this project according to the Response Assessment in Neuro-Oncology (RANO) criteria for brain metastases. In brief, partial response (PR) was defined as a 30% decrease in the longest diameter of CNS target lesions. Progressive disease (PD) was defined as a 20% increase in the longest diameter of CNS target lesions.^16^ Longest tumor diameters were assessed on T2-weighted/FLAIR images as well as on T1-weighted images after gadolinium (Gd)-contrast administration.

### Ethics Statement

Retrospective analysis of patient data was approved by local ethics committees.

### Statistics

Progression-free survival (PFS) time was defined as the time from initiation of bevacizumab-based treatment to a locally assessed radiological diagnosis of progression or death. The Kaplan–Meier product-limit method was used to estimate PFS. A two-tailed *P*-value ≤.05 was considered to indicate statistical significance. Statistical analysis was performed with Statistical Package for the Social Sciences (SPSS) 23.0 software (SPSS Inc.).

## Results

### Patient Characteristics

**Figure 1.**
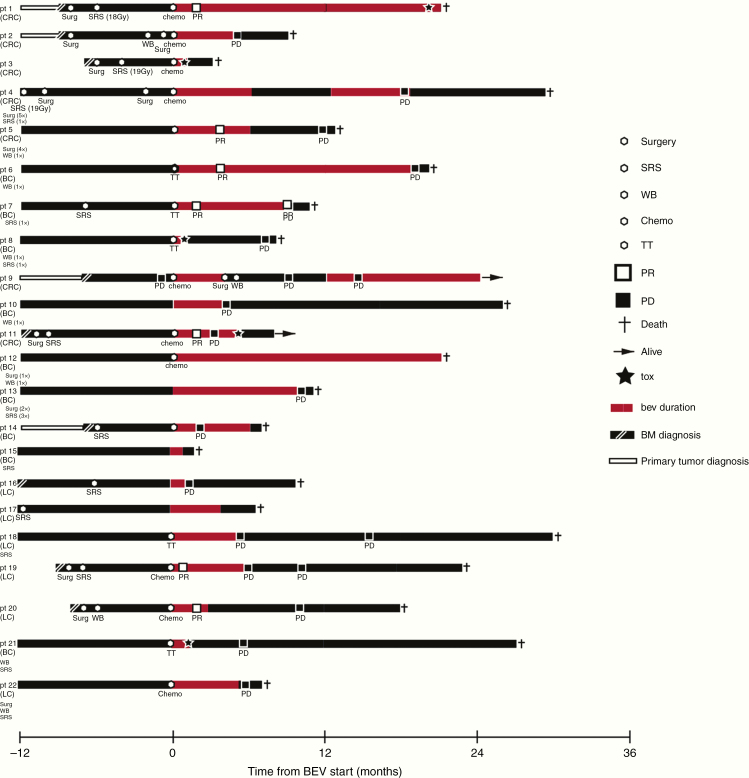
Timelines demonstrating the clinical course of disease of all included patients. Each line represents an individual patient. Study inclusion with the start of bevacizumab treatment is set to timepoint 0. Patient history is shown up to 12 months prior to study inclusion. The primary tumor is indicated on the left, and prior therapies are listed below. LC, non-small cell lung cancer; BC, breast cancer; CRC, colorectal cancer; SRS, radiosurgery; WB, whole-brain radiotherapy; TT, targeted treatment; PR, brain-specific partial response; PD, brain-specific progressive disease; tox, treatment-associated toxicity; BM, brain metastases; bev, bevacizumab.

Twenty-two patients (8 [36.4%] male; 14 [63.6%] female) with BM from breast cancer (9/22; 40.9%), colorectal cancer (7/22; 31.8%), or lung cancer (6/22; 27.3%) were included in the analysis. All included patients had at least one previous BM directed local treatment including radiosurgery in 16/22 (72.7%) patients, WBRT in 8/22 (36.4%) patients, and neurosurgical resection in 11/22 (50.0%) patients (Table 1). All patients presented with neurological symptoms in need of steroid treatment and progressive BM. The median number of BM was 1 (range 1–4), and 12/22 (57.1%) patients presented with a single BM. Systemic metastatic disease was present in 10/22 (45.4%) patients, while 12/22 (54.5%) patients presented with brain-only metastatic disease in the absence of extracranial metastases at the timepoint of bevacizumab initiation. Of 22 patients 4 (18.1%) presented with simultaneous extracranial progression. Median Karnofsky performance score was 80 (range 40–90). Median time since diagnosis of cancer and the development of BM was 15.5 months (range 0–112 months). None of the investigated patients received a radiological diagnosis of radionecrosis at the initiation of bevacizumab, although perfusion imaging was not available in all patients. However, radionecrosis can principally not been ruled out with certainty in patients who received radiosurgery 3–24 months before (63.6% in our series).^17^Table 1 lists further patient characteristics, and Figure 1 shows the timelines for every single patient.

**Table 1.** Patients Characteristics

	Entire Cohort, *n* = 22
	*n* (%)
Median age at bevacizumab start, years (range)	56 (30–75)
Gender	
Female	14 (63.6)
Male	8 (36.4)
Primary tumor type	
Breast cancer	9 (40.9)
Colorectal cancer	7 (31.8)
Lung cancer	6 (27.3)
Time since diagnosis of BM to bevacizumab start, months (range)	16.5 (4–55)
Previous BM directed treatments	
SRS	16 (72.7)
WBRT	8 (36.4)
Neurosurgical resection	11 (50.0)
Therapeutic combination partner of bevacizumab	
Any systemic combination	14 (63.6)
None (bevacizumab monotherapy)	8 (36.4)
Chemotherapy-based combination	10 (45.5)
5-Fluorouracil	3 (13.6)
5-Fluorouracil + Irinotecan	3 (13.6)
5-Fluorouracil + Oxaliplatin	1 (4.5)
Carboplatin + Paclitaxel	1 (4.5)
Carboplatin + Pemetrexed	1 (4.5)
INN-doxorubicin	1 (4.5)
Targeted therapy-based combination	3 (13.6)
Trastuzumab + Lapatinib	1 (4.5)
Anastrozol	1 (4.5)
Gefitinib	1 (4.5)
Chemotherapy + targeted therapy-based combination	1 (4.5)
Carboplatin + Paclitaxel + Gefitinib	1 (4.5)

BM, brain metastasis; SRS, stereotactic radiosurgery; WBRT, whole-brain radiotherapy.

### Bevacizumab-Based Treatment as Salvage Therapy

Of 22 patients 14 (63.6%) with symptomatic progression of BM required steroid treatment to relieve neurological symptoms. Time since diagnosis of BM to initiation of bevacizumab-based treatment was 16.5 months (range 4–55). All patients were previously pretreated with radiotherapy. The median time since the last radiotherapy was 9 months (range 2–29 months). The therapeutic approach in all patients was discussed in a multidisciplinary tumor board, which revealed that local therapy was not feasible in the included patients due to the previous radiation dosing, and lack of a meaningful option for surgical resection due to previous therapies, multifocality, and/or its eloquent location in the brain.

Of 22 patients 14 (63.6%) received concurrent systemic therapy during bevacizumab therapy (see Table 1 for details). The median time of bevacizumab-based treatment was 5 months (range 0–21). Until the last follow-up, 21/22 (95.4%) patients stopped bevacizumab-based treatment. Here, progression was the most frequent reason to stop in 9/21 (42.9%) patients.

No unexpected side effects were observed. Of 21 patients 5 (23.8%) stopped bevacizumab-based treatment due to side effects. Wound healing problems were observed in 3/22 (13.6%) patients, intracerebral hemorrhage in 1/22 (4.5%) patients (where it was fatal), and a bowel fistula in 1/22 (4.5%) patients. Of 21 patients 7 (33.3%) discontinued bevacizumab treatment due to patient wish (2/7, 28.6%) or as a consented treatment break after stabilization of symptoms over 3 months (5/7, 71.4%).

### Responses to Bevacizumab-Based Therapy

Clinically, 14/22 (63.6%) patients experienced improvement of neurological symptom burden, and in 6/22 (27.3%) patients the neurological symptom burden could be stabilized (Table 2). In line, steroid dosing could be lowered or omitted in 15/22 (68.2%) patients. Stabilization of steroid dosing was achieved in 7/22 (31.8%) patients. Of 22 patients 2 (9.1%) experienced further neurological deterioration under the initiated bevacizumab-based therapy. In consequence, clinical benefit was observed in 20/22 (90.9%) patients after the initiation of a bevacizumab-based treatment. The clinical benefit rate in 8 patients receiving bevacizumab monotherapy was 100% as all patients had either stabilization of symptoms (4/6, 66.7%) or improvement (2/6, 33.3%). Of 8 patients 2 (25.0%) receiving bevacizumab-based monotherapy had previously received WBRT only.

**Table 2.** Response Assessment to Bevacizumab-Based Treatment

	Entire Cohort, *n* = 22
	*n* (%)
Best clinical response to bevacizumab-based treatment	
Stable disease	6 (27.3)
Improvement	14 (63.6)
Progressive disease	2 (9.1)
Best radiological intracranial response to bevacizumab-based treatment (T2/FLAIR); *n* = 20	
Stable T2/FLAIR	8 (40.0)
Improvement of T2/FLAIR (>25%)	6 (30.0)
“Significant” decrease of T2/FLAIR (>50%)	6 (30.0)
“Significant increase” of T2/FLAIR (>25%)	0 (0.0)
Best radiological intracranial response to bevacizumab-based treatment based on RANO-BM (T1 GBCA [Gd-based contrast agent]); *n* = 19	
Stable disease	9 (47.3)
Partial response	7 (36.8)
Progressive disease	3 (15.7)
Reduction of steroid treatment	
Yes	15 (68.2)
No	7 (31.8)
Reason for determination of bevacizumab-based treatment	
Progression	9 (40.9)
Toxicity	5 (22.7)
Other/Unclear	7 (31.8)
On-going	1 (4.5)
Extracranial progression after bevacizumab-based treatment	
Yes	11 (50.0)
No	11 (50.0)
Intracranial progression after bevacizumab-based treatment	
Yes	15 (68.2)
No	7 (31.8)
Alive at last follow-up	
Yes	3 (13.6)
No	19 (86.4)
Median time to intracranial progression or death, months (range)	8 (1–24)
Median overall survival time from the start of bevacizumab-based treatment, months (range)	17 (1–43)

Clinical improvement after bevacizumab-based treatment was not associated with concurrent chemotherapy, type of primary tumor, GPA class, or presence of extracranial disease (*P* > .05). Due to the small sample size no additional correlation between the single systemic therapy regimen and clinical improvement was calculated.

**Figure 2.**
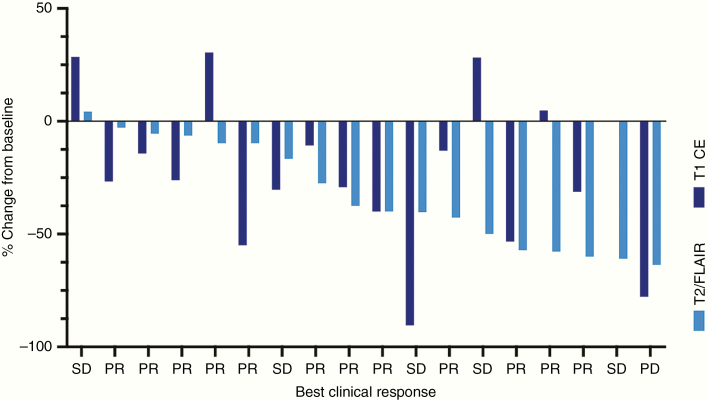
Waterfall plot demonstrating the intracranial response to bevacizumab-based treatment in patients with recurrent BM on T1 CE (after Gd-contrast enhancement) and T2/FLAIR MRI images. FLAIR sequence or if not available T2 sequence was used for response assessment. The best clinical response for each patient is given below. SD, stable disease; PR, partial response; PD, progressive disease.

**Figure 3.**
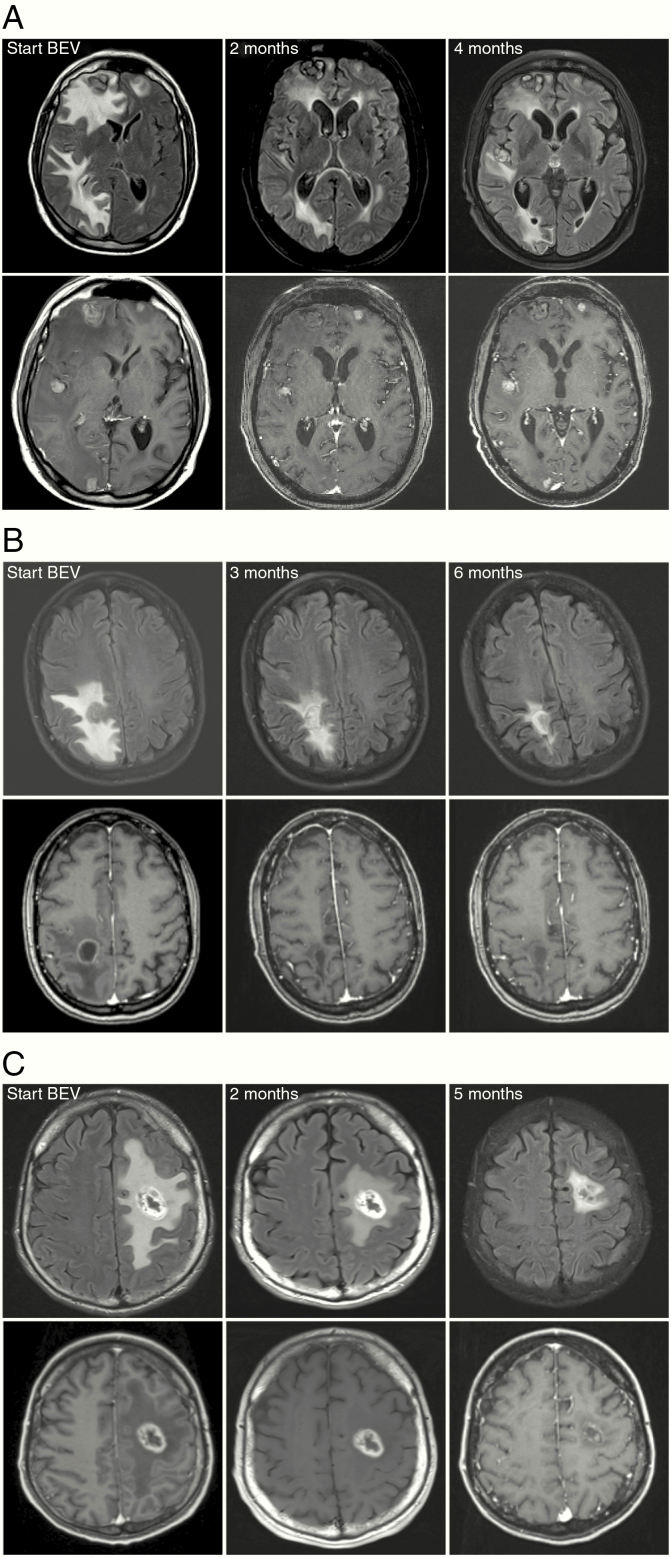
(A) MR images of baseline (BL) and follow-up MRI of a lung cancer patient with a partial response after initiation of bevacizumab-based treatment. Partial response is seen both on FLAIR (upper row) and T1-weighted (lower row) images. (B and C) MR imaging examples of patients with breast cancer (B) and colorectal cancer (C) showing partial response on FLAIR and complete response on T1-weighted images after Gd-contrast administration (B) on follow-up MRI. Note that the tumor volume (central core) and perifocal edema can be differentiated on FLAIR images.

Pre- and posttreatment MRI images were available in 20/22 (90.9%) patients for T2-weighted/FLAIR and in 19/22 (86.3%) patients for T1-weighted after Gd-contrast agent administration. The median best response for T1-weighted after Gd-contrast agent administration was −26.0% from baseline (range −90.5% to 30.4%; Figure 2). According to RANO-BM criteria for T1-weighted after Gd-contrast agent administration, 9/19 (47.3%) patients presented with stable disease and 7/19 (36.8%) showed PR as best imaging response on follow-up imaging (Figure 2). Of 19 patients 3 (15.7%) presented with PD as investigated by T1-weighted after Gd-contrast agent administration. Repetitive MRI images over time were available for 1/2 (50.0%) patients with neurological deterioration under bevacizumab-based treatment, 4/6 (66.6%) with clinical stabilization, and 12/14 (85.7%) with clinical improvement. The one patient with neurological deterioration actually presented with partial response in T1-weighted and T2/FLAIR imaging. About 66.7% (2/3) patients with progression on T1-weighted imaging as defined by RANO criteria actually presented with a stable clinical burden and 33.3% (1/3) even with an improvement of neurological symptoms despite radiological progression. Exemplary MRI images of 3 responding patients are shown in Figure 3. In primary brain tumors, the tumor border is often difficult to delineate, and “pseudoresponse” phenomena under bevacizumab treatment have been widely discussed. In BM, however, the tumor–brain border is clearer in T1- and T2-weighted images and allows to better delineate the extent of the antitumor effect of bevacizumab in comparison to an evaluation in primary brain tumors. As shown in Figure 3, “true” tumor responses can be radiologically suspected in this patient cohort.

**Figure 4.**
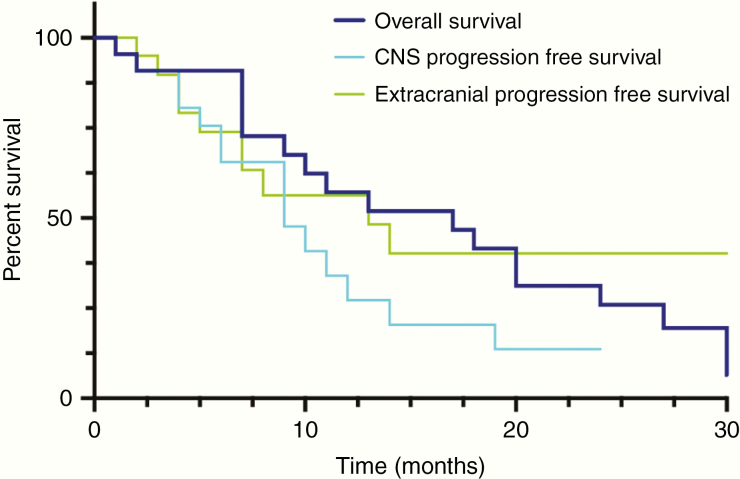
Kaplan–Meier plot showing overall survival, CNS-specific progression-free survival, and extracranial progression-free survival after initiation of bevacizumab-based treatment.

Of 22 patients 15 (68.2%) experienced subsequent CNS progression and 11/22 (50.0%) systemic progression after initiation of bevacizumab-based treatment. The likelihood of systemic progression or CNS progression did not correlate with the addition of chemotherapy to bevacizumab-based treatment (>0.05). CNS-specific PFS was 8 months (range 1–24 months) and systemic specific PFS was 13 months (range 0–30; Figure 4). Median overall survival after initiation of bevacizumab-based treatment was 17 months (range 1–43 months; Figures 1 and 4).

## Discussion

Bevacizumab-based treatment had high clinical efficacy in the current cohort of patients with symptomatic, progressive brain metastatic disease without any meaningful options for further local treatment. Given the longer survival of BM patients due to the improvements of local and systemic treatments, an increasing number of patients experience perseverative, symptomatic recurrence of BM with high neurological sequelae and the need for high steroid doses.^1^ Therefore, the development of additional treatment options for this patient cohort is urgently needed.^3,18,19^ The results from this case series underscore the value of bevacizumab-based treatment as a well-tolerated, clinically meaningful salvage treatment option for patients with recurrent, symptomatic BM and no local or other meaningful treatment options.

The included patients all suffered from a progression of previously treated BM and had no possibility of additional local radiotherapy due to the high risk of subsequent neurotoxicity, including leukodystrophy and radionecrosis.^20^ Patients included in our study were mostly on high steroid treatment to control the symptomatic burden. Generally, the symptomatic relief provided by steroids is of a transient nature, and side effects are very frequent, including insomnia, myopathy, psychiatric side effects, and Cushing syndrome which all further negatively impact the quality of life.^7^ Therefore, alternative treatment modalities to control peritumoral edema and mass effect are urgently warranted: in addition to the unfavorable side effect profile, steroids have no anti-neoplastic effect, and high steroid dosing is actually associated with poor overall survival in primary brain tumor patients.^21^ We observed a considerably long survival in the present patient cohort of 17 months from the start of bevacizumab-based treatment. Furthermore, clinical response was also observed in patients with only radiological stabilization or even progression of BM, underscoring that the strong anti-edema effect of bevacizumab can have high clinical efficacy in BM patients. Given the median survival of 7–9 months in an unselected cohort of BM patients, we cannot however exclude an inclusion bias in our case series^22^ consisting mainly of patients with oligometastatic disease. Nevertheless, adequate symptom control in the absence of fast-progressing disease might be of even higher clinical importance, as BM are always incurable and optimal symptom control needs to be a leading clinical goal.

Bevacizumab-based treatment resulted in an objective radiological response as measured by the RANO criteria^16^ and also a clinical benefit in the vast majority of patients. Surprisingly, no strong linear correlation between the radiological response and the clinical benefit was observed. In support of the findings reported in this study, bevacizumab has shown tumor-specific activity in combination with cytotoxic agents in BM. The BRAIN trial investigated in a single-arm study the combination of platin-based chemotherapy plus bevacizumab in patients with newly diagnosed, asymptomatic BM from non-small cell lung cancer.^23^ Here, the intracranial response rate was even 61.2% in comparison to an extracranial response rate of 64.2%. However, the impact on symptomatic control was less clear in this study.^23^ Furthermore, intracranial responses were also shown in breast cancer and colorectal cancer patients suffering from BM, suggesting a potential benefit of bevacizumab for all main tumor entities frequently causing BM with the exception of melanoma.^15,24,25^ In glioblastoma, bevacizumab did not result in an overall survival benefit if added to standard treatment but in symptomatic improvement, reduction of steroid treatment, and improvement of PFS.^26–28^ Therefore, the true antiproliferative effect of bevacizumab might be limited in comparison to clinically relevant symptom control.^7^ Given the current lack of established therapies in recurrent BM, antiangiogenic therapies are therefore a valuable armamentarium to the treatment of recurrent symptomatic BM.

Bevacizumab-based treatment was shown to be a safe salvage treatment option. Although bevacizumab was initially associated with intracranial hemorrhage based on the report of a single case of a patient suffering from BM from liver carcinoma, several consequent large follow-up analyses showed a good safety profile in patients with BM receiving bevacizumab.^23,29^ In our cohort, one patient suffered from intracranial hemorrhage and subsequent death. Certainly, bevacizumab-based treatment has to be applied with caution and needs to be critically discussed in patients with a history of intracranial bleeding. However, the otherwise favorable safety profile, especially in comparison to long-term high dosing of steroids, underscores the clinical utility as a salvage treatment in BM patients.

Limitations of our investigation include the limited number of patients as well as the retrospective design of the study. Therefore, the favorable survival prognosis in the present cohort might be impacted by an inclusion and selection bias. Also, there was no uniform treatment schedule, as different bevacizumab dosing and different systemic therapy combinations were applied. Dosing schedules differed according to the primary tumor type, as the recommended dosing for non-small cell lung cancer is 7.5 mg/kg or 15 mg/kg every 3 weeks, for breast cancer 10 mg/kg every 2 weeks or 15 mg/kg every 3 weeks, and for colorectal cancer 7.5 mg/kg every 2 weeks or 15mg/kg every 3 weeks. So far none of these licensed dosing schedules was shown to be superior. Indeed, the dosing of 400 mg every 2 weeks irrespective of bodyweight was initiated in glioblastoma treatment due to practical reasons as one serving bevacizumab includes 400 mg, preventing the discard of costly bevacizumab. Similar clinical efficacy of low-dose and high-dose bevacizumab was observed in glioblastoma patients, supporting the cost-adapted dosing of 400 mg every 2 weeks that was also applied in some patients in the current study.^30^ Furthermore, given the sometimes short interval between the last radiation, some patients might have suffered from radionecrosis masking as tumor progression. Nevertheless, bevacizumab has been shown to be radiologically and, more importantly, clinically effective in the treatment of radionecrosis in BM patients, and the data of our present cohort further support the palliative value of bevacizumab.^31–34^

In conclusion, this case series strongly suggests that bevacizumab-based treatment is a clinically effective and well-tolerated salvage treatment in patients with progressive symptomatic BM and no remaining local and limited other systemic treatment options. The potential side effects of bevacizumab-based treatment such as hemorrhage, bowel perforation, deep vein thrombosis, or pulmonary embolism certainly require adequate patient selection and monitoring. Importantly, the apparent antitumor activity of bevacizumab specifically in the context of BM as supported by this study, in combination with its proven beneficial effects in radiation necrosis, makes this class of drugs a plausible choice for such heavily pretreated patients.^15^ This is particularly the case if steroids fail to improve clinical symptoms, or if steroid dependency is chronically high leading to accumulating side effects. In consequence, we consider this retrospective analysis with its promising results as a reasonable foundation for a future controlled clinical trial testing bevacizumab in a larger patient population with symptomatic, pretreated BM.

## Notes

**Authorship Statement.** Study design, data collection, data interpretation, manuscript writing, and approval of final manuscript version: A.S.B., M.O.B., L.R., K.K.J., S.L., and F.W.

Study design, data collection, data interpretation, and approval of final manuscript version: S.L., F.S., J.F., M.C., M.T., M.J.S., F.M., L.L.M., T.S., D.J., M.B., M.P., and W.W.
